# An Evaluation of the Most Convenient and Effective Procedure for Reducing the Loading Time of Epirubicin Into the Drug-Eluting Bead M1 (DC Bead M1™)

**DOI:** 10.7759/cureus.72352

**Published:** 2024-10-25

**Authors:** Yusuke Kawamura, Norio Akuta, Shigeki Yamamoto, Yasuka Eriksson, Tetsuya Hosaka, Satoshi Saitoh, Hitomi Sezaki, Fumitaka Suzuki, Kenji Ikeda, Hiromitsu Kumada

**Affiliations:** 1 Hepatology, Toranomon Hospital, Tokyo, JPN; 2 Okinaka Memorial Institute for Medical Research, Toranomon Hospital, Tokyo, JPN

**Keywords:** advanced hepatocellular carcinoma, dc bead m1, drug-eluting bead, epirubicin, systemic therapy

## Abstract

Background and objective

Drug-eluting beads (DEB) have been highly useful in the current treatment strategies for multiple and large hepatocellular carcinomas (HCC) with or without systemic therapy. Recently, smaller beads have become available in Japan. In this study, we aimed to evaluate the most convenient and effective way to reduce the loading time of epirubicin into the drug-eluting beads M1 (DC Bead M1^TM^; 70-150 µm).

Methods

To reduce the loading time of epirubicin into DC Bead M1, we used a method that involved mixing the drug and then agitating the solution using vortexing: Group A: agitated for 15 sec; Group B: agitated for 20 sec; Group C: agitated for 30 sec; and Group D: left at room temperature as the control group. After the loading of epirubicin by each method, the supernatant concentration of epirubicin was assayed at 5, 10, 15, 20, 30, 60, and 120 min.

Results

Epirubicin loading rates for DC Bead M1 at five min were 99.7% in Group A, 98.7% in Group B, 99.6% in Group C, and 99.5% in Group D. The four groups reached an equilibrium between five and 120 min. Surprisingly, Group D (left at room temperature for five min) showed the same level of epirubicin loading rate compared to that of Groups A, B, and C at five min (*p*=0.566). Morphological analysis showed that there were no significant morphological changes for each agitated time up to 30 sec compared with that observed for the beads left at room temperature.

Conclusions

The most convenient and effective way for reducing the loading time of epirubicin into DC Bead M1 was observed in Group D (left at room temperature for five min).

## Introduction

Hepatocellular carcinoma (HCC) is the most common form of liver cancer, and the third most common form of cancer [1]. And, the Barcelona Clinic Liver Cancer (BCLC) system currently recommends transarterial chemoembolization (TACE) for the treatment of intermediate-stage HCC [2]. However, in the current treatment strategy for HCC, TACE is often performed in combination with systemic therapy to control intrahepatic lesions in the intermediate (stage B) and advanced stages of BCLC stage C [3-7]. In addition, TACE using drug-eluting beads (DEB; DEB-TACE) is attracting attention as a useful combination of systemic therapy and TACE [4,5,8]. A synergistic effect with immunotherapy is also expected to occur [9].

In general, smaller diameter beads are recommended in TACE for HCC [10], and until now, small beads (100-300 µm) have been mainly used in Japan. However, since April 2024, smaller beads (DC Bead M1^TM^; 70-150 µm) have been available in Japan, and these beads may become the main type used in DEB-TACE for HCC in the future. In Japan, epirubicin (Farmorubicin^TM^, Pfizer Japan, Tokyo, Japan) is used commonly instead of doxorubicin to load into DC Bead^TM^ due to the cardiac side effects of doxorubicin [11,12]. However, the loadability of epirubicin into DC Beads and ways to shorten the loading time have not yet been fully elucidated. We have previously reported on the loadability of epirubicin into DC Bead and also a method for reducing the loading time of epirubicin into these beads by shaking the vial on a vortex mixer [13]. In addition, to date, there is no data on the loadability of doxorubicin into DC Bead M1 for less than 30 min [14].

The current study was similar to our previous investigation but used smaller diameter beads (DC Bead M1; 70-150 µm) and involved basic research on the effect of changes in terms of using a particle size on the loading time of epirubicin and the impact on bead shape following agitation with a vortex mixer.

## Materials and methods

Embolization microsphere DC Bead M1: product information

The embolization microsphere DC Bead M1^TM^ (Boston Scientific, Marlborough, MA) is a drug-eluting bead capable of loading and releasing chemotherapeutic agents in a controlled manner. DC Bead M1 was supplied in sterile vials as a 2 mL hydrated bead volume in 6 mL of phosphate-buffered saline (PBS).

Antitumor agent and loading into the DC Bead M1

**Figure 1:**
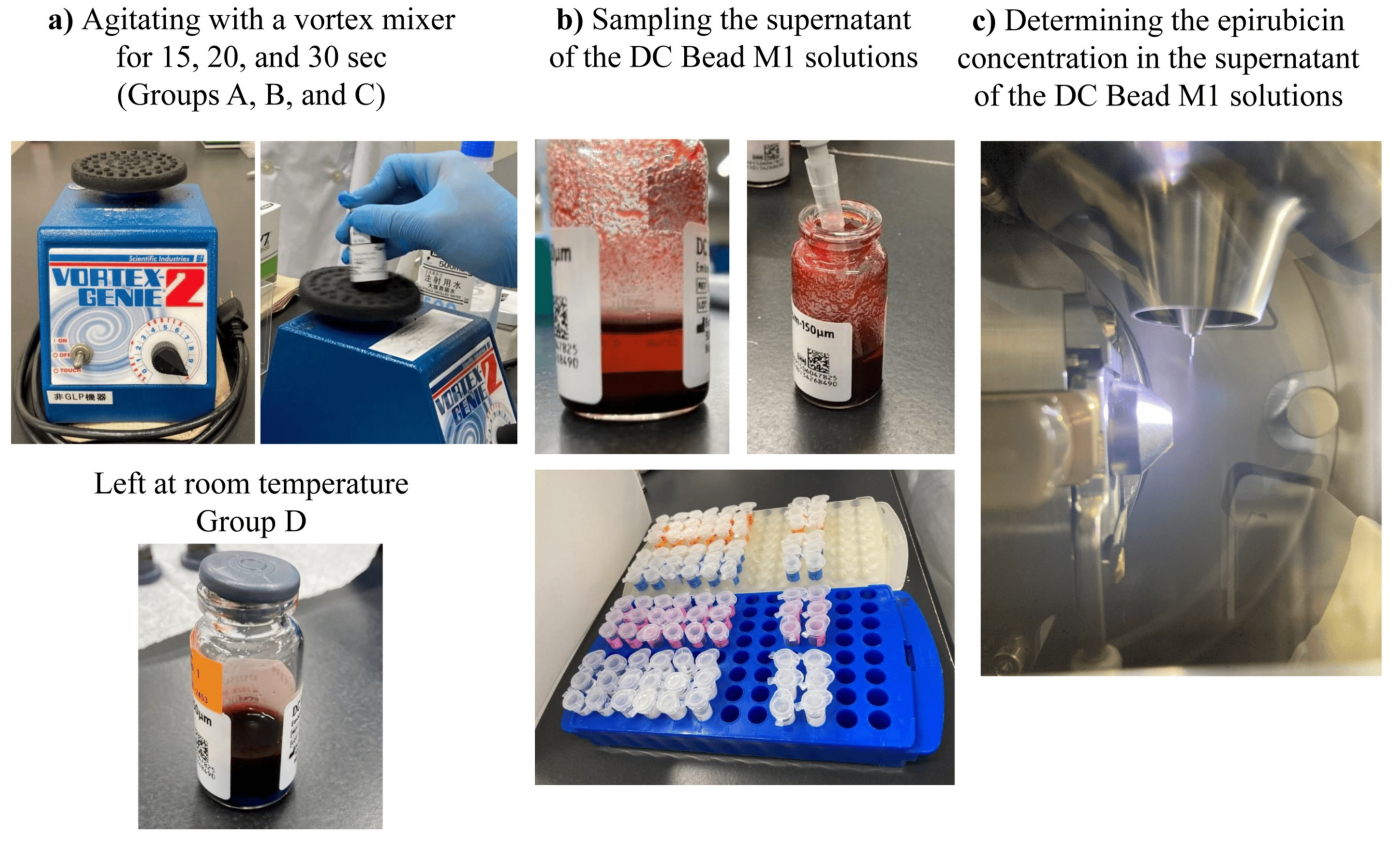
Procedures in the loading of epirubicin into DC Bead M1 a) Agitating with a vortex mixer for 15, 20, and 30 seconds (Groups A, B, and C) or left at room temperature without agitation using a vortex mixer (Group D - control). b) Sampling the supernatant of the DC Bead M1 solution. c) Determining the epirubicin concentration in the supernatant using a triple quadrupole mass spectrometer

Epirubicin (Farmorubicin^TM^, Pfizer Japan, Tokyo, Japan) is a highly water-soluble anti-tumor agent used for the treatment of HCC. A total of 50 mg of epirubicin was reconstituted in 2 mL of water for injection (Otsuka Pharmaceutical, Tokyo, Japan). A 6 mL aliquot of PBS was removed from the vial of DC Bead M1, followed by the addition of 2 mL of reconstituted epirubicin solution. The DC Bead M1 and epirubicin solution was then agitated using a vortex mixer (Vortex-Genie 2 Scientific, Scientific Industries, Philadelphia, PA) for either 15 sec (Group A), 20 sec (Group B), 30 sec (Group C), or left at room temperature without agitation using a vortex mixer (Group D - control) (Figure 1). We examined the four types of epirubicin loading in triplicate to confirm the reproducibility of each procedure.

Assessment of epirubicin loading

After the addition of the epirubicin solution, the loading of the drug into DC Bead M1 was assessed at 5, 10, 15, 20, 30, 60, and 120 min in each of the four groups. The ratio of drug uptake into the beads was calculated from the residual unloaded drug content in the solution measured using a triple quadrupole mass spectrometer (Xevo^TM^ TQ Absolute, WatersTM, Milford, MA) equipped with an electrospray ion source in the positive mode [15-17]. Quantitation was performed using multiple reaction monitoring (MRM) with a dwell time of 30 ms per transition. The supernatant concentration of epirubicin in the four groups was measured in triplicate, with the mean epirubicin concentrations applied to make the drug loading profiles.

Morphology of the microspheres

A morphological assessment of the triplicate DC Bead M1 samples collected after loading was performed in the four groups by using a digital microscope (EZ4-CAM106, Sanwa Supply Inc, Okayama, Japan).

Statistical analysis

The statistical analyses were performed using IBM SPSS Statistics software ver. 30.0 (IBM Corp., Armonk, NY). The data were expressed as mean and standard deviation (SD). Differences in the supernatant concentration and loading rate of epirubicin in each group were assessed using one-way ANOVA. A p-value<0.05 was considered to denote a statistically significant difference.

## Results

Loading rate of epirubicin into DC Bead M1

Table 1 summarizes the epirubicin concentrations in the supernatant of the DC Bead M1 solution.

**Table 1:** Epirubicin concentration in the supernatant of DC Bead M1 solutions (ug/mL)* ^*^Mean (n=3 in each procedure). ^†^Before agitation

	Time (min)							
Agitated time with the vortex mixer and epirubicin concentration (µg/mL)	0^†^	5	10	15	20	30	60	120
15 sec	22,400.0	72.9	144.0	103.0	72.4	87.7	67.7	77.4
20 sec	21,600.0	274.0	316.0	186.0	177.0	260.0	251.0	98.6
30 sec	20,800.0	78.3	95.0	126.0	97.8	94.9	64.7	68.2
0 sec (left at room temperature: control)	21,500.0	111.0	63.1	77.8	78.7	45.0	77.5	84.7

For the vortex mixing times of 15 sec (Group A), the epirubicin concentrations remaining in the supernatant of the microsphere solution were 22,400.0 µg/mL at zero min (i.e., before agitation), 72.9 µg/mL at five min, 144.0 µg/mL at 10 min, 103.0 µg/mL at 15 min, 72.4 µg/mL at 20 min, 87.7 µg/mL at 30 min, 67.7 µg/mL at 60 min, and 77.4 µg/mL at 120 min. For the vortex mixing time of 20 sec (Group B), the epirubicin concentrations remaining in the supernatant of the microsphere solution were 21,600.0 µg/mL at zero min (before agitation), 274.0 µg/mL at five min, 316.0 µg/mL at 10 min, 186.0 µg/mL at 15 min, 177.0 µg/mL at 20 min, 260.0 µg/mL at 30 min, 251.0 µg/mL at 60 min, and 98.6 µg/mL at 120 min.

For the vortex mixing time of 30 sec (Group C), the epirubicin concentrations remaining in the supernatant of the microsphere solution were 20,800.0 µg/mL at zero min (before agitation), 78.3 µg/mL at five min, 95.0 µg/mL at 10 min, 126.0 µg/mL at 15 min, 97.8 µg/mL at 20 min, 94.9 µg/mL at 30 min, 64.7 µg/mL at 60 min, and 68.2 µg/mL at 120 min. Finally, without vortexing and left at room temperature (Group D, control), the epirubicin concentrations remaining in the supernatant of the microsphere solution at zero min were 21,500.0 µg/mL (before agitation), 111.0 µg/mL at five min, 63.1 µg/mL at 10 min, 77.8 µg/mL at 15 min, 78.7 µg/mL at 20 min, 45.0 µg/mL at 30 min, 77.5 µg/mL at 60 min, and 84.7 µg/mL at 120 min.

The epirubicin loaded into DC Bead M1 was calculated as the original amount of epirubicin in the supernatant minus the amount of epirubicin in the supernatant after loading into the beads (Table 2).

**Table 2:** Epirubicin loading rate into DC Bead M1 (%)* ^*^Mean (n=3 in each procedure). ^†^Before agitation

	Time (min)							
Agitated time with the vortex mixer and loading rate of epirubicin (%)	0^†^	5	10	15	20	30	60	120
15 sec	0	99.7	99.4	99.5	99.7	99.6	99.7	99.7
20 sec	0	98.7	98.5	99.1	99.2	98.8	98.8	99.5
30 sec	0	99.6	99.5	99.4	99.5	99.5	99.7	99.7
0 sec (left at room temperature: control)	0	99.5	99.7	99.6	99.6	99.8	99.6	99.6

**Figure 2:**
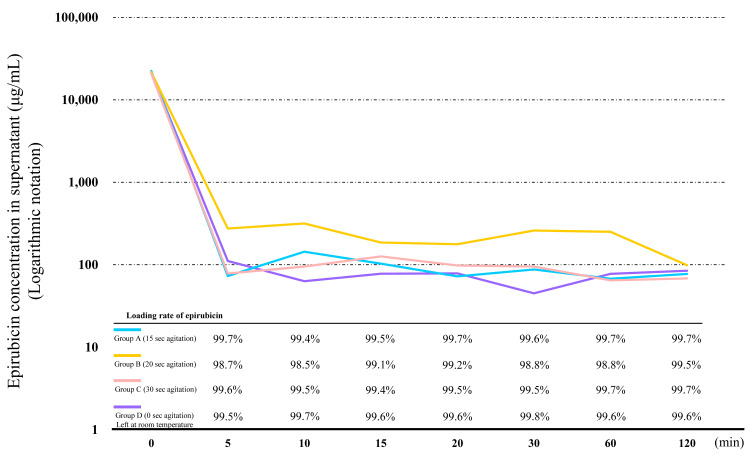
Concentration and loading rate of epirubicin in DC Bead M1 (70-150 µm) for each mixing time using a vortex mixer Agitated for 15 sec (Group A), agitated for 20 sec (Group B), agitated for 30 sec (Group C), or left at room temperature (Group D)

The loading rate was then calculated for the four groups (Figure 2). The loading rates of epirubicin for DC Bead M1 at five min were 99.7% in Group A, 98.7% in Group B, 99.6% in Group C, and 99.5% in Group D. The four groups all reached an equilibrium between 5 and 120 min. There were no significant differences in the epirubicin concentration and loading rate between each group at five min (*p*=0.638 and 0.566, respectively).

Morphological analysis of DC Bead M1 after agitation using a vortex mixer

**Figure 3:**
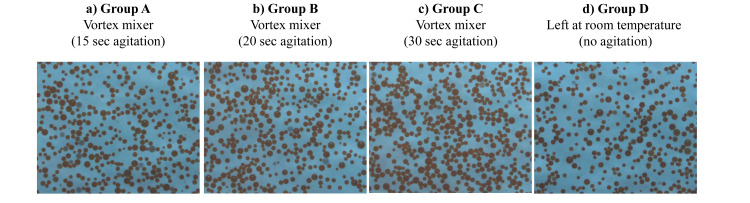
Morphological changes of DC Bead M1 at various agitated times using a vortex mixer* a) agitation for 15 sec (Group A), b) agitation for 20 sec (Group B), c) agitation for 30 sec (Group C), or d) left at room temperature (no agitation; Group D). Agitation for 30 sec did not affect the microscopic findings Morphological examination was performed one week after using the preserved sample. Original magnifications: a) ×100; b) ×100; c) ×100; and d) ×100

Figure 3 shows the morphological changes of DC Bead M1 at different agitation times. There were no significant morphological changes in each agitated time up to 30 seconds (Groups A, B, and C) compared with that observed in the group left at room temperature (Group D).

## Discussion

DC Bead is composed of a polyvinyl alcohol polymer modified with sulfonate groups to form a hydrogel with a high water content (>95%). The negatively charged sulfonate interacts with the positively charged protonated amine groups of doxorubicin hydrochloride via an ion exchange process driven by entropic release of the smaller counter ions from the hydrogel [18]. Preclinical and clinical studies have shown higher and prolonged retention of doxorubicin within the tumor after TACE using DC Bead, and lower systemic plasma levels of doxorubicin, compared to that achieved by conventional TACE (cTACE) [19-24]. A randomized controlled trial - the PRECISION V study- demonstrated that TACE using DC Bead had a higher response rate than that of conventional TACE using lipiodol and varied embolic substances, although this was not statistically significant [25]. On the other hand, the inferiority of DEB-TACE compared to cTACE has been reported recently [26]. These divergences in the therapeutic efficacy of DEB-TACE may be addressed with the advent of the smaller-diameter DC Bead M1.

We have reported previously that 30 sec of agitation of DC Bead (100-300 µm) using a vortex mixer shortens the loading time of epirubicin, and this method is now used widely in clinical practice in Japan. Now that DC Bead M1 is newly available, we conducted basic research on the effect of the change in particle size on the loading time of epirubicin and the impact of agitation using a vortex mixer on bead shape. Firstly, the loading rate of epirubicin was surprisingly high, with 99.5% achieved within five min even when the beads were left at room temperature, with the results comparable to those obtained using a vortex mixer. Previous reports have only examined loading rates after 30 min [14], and therefore the results of the current study will have a significant impact on the use of DC Bead M1 in clinical practice.

Based on the product information of DC Bead M1, the number of particles per vial of DC Bead is 200,000 for 100-300 µm and 1,300,000 for 70-150 µm. As a result, theoretically, the surface area of DC Bead M1 (70-150 µm) is significantly greater than that of the 100-300 µm beads, which could explain how such a high loading rate of 99.5% is achieved when the beads are only left at room temperature for five min. No significant morphological changes were observed in the beads in the four groups. Based on the results of this study and considering the simplicity of use in actual clinical practice, we conclude that the best method for loading epirubicin into DC Bead M1 involves leaving the solution at room temperature for five min.

This study had a relatively small sample size, which constitutes a major limitation. Hence, we plan to carry out further research with a larger sample size shortly.

## Conclusions

In our study, each of the four groups showed a similar loading ability regarding the loading of epirubicin into DC Bead M1. Hence, the vial-shaking did not affect the loading ability of epirubicin into DC Bead M1 at five min. The most convenient and effective way for reducing the loading time of epirubicin into DC Bead M1 was observed in Group D (left at room temperature for five min).
